# The Effects of a Chactoid Scorpion Venom and Its Purified Toxins on Rat Blood Pressure and Mast Cells Histamine Release

**DOI:** 10.3390/toxins5081332

**Published:** 2013-07-29

**Authors:** Keren Ettinger, Gadi Cohen, Tatjana Momic, Philip Lazarovici

**Affiliations:** Institute for Drug Research, School of Pharmacy, Faculty of Medicine, The Hebrew University of Jerusalem, POB 12065, Jerusalem 91120, Israel; E-Mails: keren.ettinger@gmail.com (K.E.); gadifco@gmail.com (G.C.); momict@gmail.com (T.M.)

**Keywords:** chactoid scorpion, *Scorpio maurus palmatus*, venom, phospholipase A_2_, neurotoxin, blood pressure, histamine release

## Abstract

The effect of the venom of the Chactoid family of scorpions on blood pressure was scantly investigated and was addressed in the present study using the venom of the Israeli scorpion, *Scorpio maurus palmatus*. Blood pressure in rats was monitored via cannulated femoral artery, while venom and toxins were introduced into femoral vein. Venom injection elicited a biphasic effect, expressed first by a fast and transient hypotensive response, which lasted up to 10 min, followed by a hypertensive response, which lasted up to one hour. It was found that these effects resulted from different venom components. Phospholipase A_2_ produced the hypotensive effect, while a non-enzymatic neurotoxic polypeptide fraction produced the hypertensive effect. Surprisingly, the main neurotoxic polypeptide to mice had no effect on blood pressure. *In vitro* experiments indicated that the hypertensive factors caused histamine release from the peritoneal mast cells, but this effect is assumed to be not relevant to their *in vivo* effect. In spite of the cytotoxic activity of phospholipase A_2_, it did not release histamine. These findings suggest that the effects of venom and isolated fractions on blood pressure parameters are mediated by different mechanisms, which deserve further pharmacological investigation.

## 1. Introduction

Scorpions are venomous arthropods, members of the *Arachnida* class and the order, *Scorpiones*. They are found in all continents, including Israel. The scorpion species that are medically important in Israel belong to the families of Buthidae and Scorpionidae, represented by the genera, *Leiurus* *quinquestriatus quinquestriatus* and *Scorpio maurus palmatus*. The envenomation by these scorpions is considered a public and veterinary health problem in Israel, as in other countries [1]. The clinical symptoms upon envenomation indicate a general stimulation of the autonomic, somatic and peripheral nervous systems [2]. The severity of scorpion envenomation and the increased risk of mortality, especially among children, is mainly attributed to cardio-respiratory pathology, therefore stimulating investigations on the effect of scorpion venoms and derived toxins on the hemodynamic perturbations in different animal models [3,4,5,6].

Earlier studies indicated that the venom of *Scorpio maurus palmatus* consists of three main groups of polypeptides: neurotoxins, which inhibit neuronal ionic channel conductance [7,8], phospholipases A_2_ [9] and cytotoxins [7]. The toxicity of the venom results from synergistic interactions between these groups of components [9,10]. However, the effect of venom and isolated compound on the blood pressure was not yet investigated and, therefore, addressed in the present study in a rat model.

## 2. Results

### 2.1. The Effect of *Scorpio maurus palmatus* Venom on Blood Pressure

**Figure 1:**
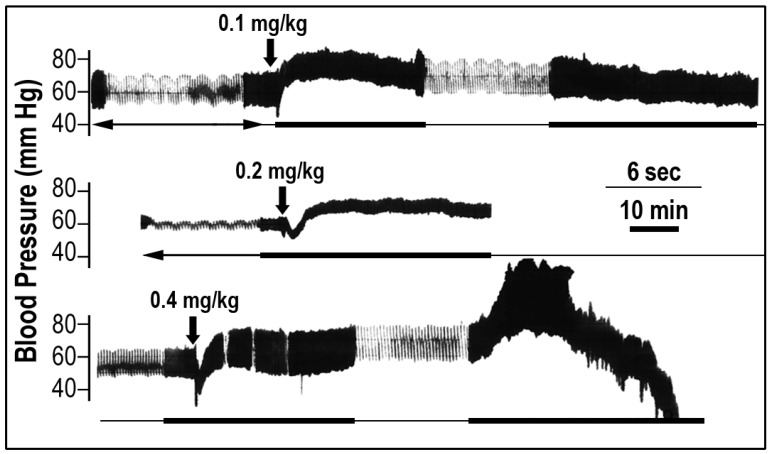
The typical effect of *Scorpio maurus palmatus* venom on blood pressure. The original tracing of the experiments show the blood pressure response (mm Hg) after addition of different doses of venom indicated by the vertical arrow. Each trace represents a single rat receiving a single dose of venom (representing measurements of eight rats). The length of the horizontal lines represents the time course of the experiment: thin line (sec); thick line (min).

Low doses of the chactoid scorpion venom injected intravenously elevated the mean arterial blood pressure from 60 ± 5 to 80 ± 5 mm Hg (*n* = 18; *p* < 0.05) (Figure 1, upper part). This hypertensive effect was dose-dependent over the doses of 0.01–0.10 mg/kg (*n* = 9 for each dose), continued for some 50–80 min and the blood pressure returned to its control values. At higher doses (0.2 mg/kg), the venom first elicited a short hypotensive effect, which lasted 5–7 min, and then, a hypertensive effect was observed (*n* = 9) (Figure 1, middle part) similar to the lower dose effect. Such a biphasic action was also seen following injection of a lethal dose (0.4 mg/kg; *n* = 6). Interestingly, the blood pressure returned to control values (60 mm Hg), then a second and violent burst of hypersensitivity (up to 120 mm Hg), which lasted approximately 10 min, occurred. During these events, skeletal muscle convulsions accompanied with sporadic respiratory apneas and heart arrhythmias appeared, then the blood pressure dropped and animal death occurred (Figure 1, lower part).

### 2.2. Chromatographic Separation of the Venom and Identification of Fractions Responsible for Blood Pressure Perturbation

**Figure 2:**
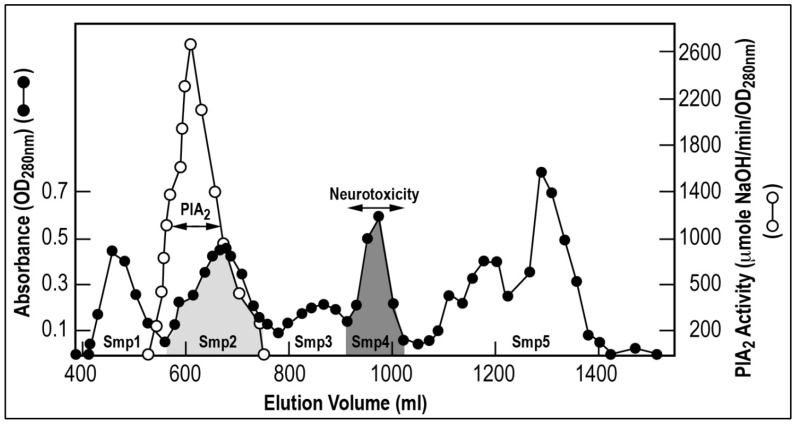
Gel filtration chromatography of *Scorpio maurus palmatus* venom. 500 mg of venom were separated on Sephadex G-50 gel equilibrated and eluted by 0.1 M ammonium acetate, pH 8.5. The flow rate was 15 mL/h, and fractions of 10 mL were collected. The marked areas correspond to PlA_2_ and neurotoxic fractions, respectively. Full circles represent protein absorbance at 280 nm, and open circles correspond to the PlA_2_ activity.

In order to identify the venom proteins responsible for the perturbation of the blood pressure, the *Scorpio maurus palmatus* (Smp) venom was separated by column chromatography on Sephadex G-50 gel (Figure 2) [7,9]. This chromatographic separation generated several protein fractions: Smp1, Smp2, Smp3, Smp4 and Smp5. Phospholipase A_2_ (PlA_2_) was detected in the Smp2 fraction, while neurotoxicity to mice and rats was detected in the Smp4 fraction. The Smp3 fraction was cytotoxic to cell cultures of sympathetic neurons (PC12 cells) and hemolytic to human red blood cells (data not shown) (Figure 2). 

**Figure 3:**
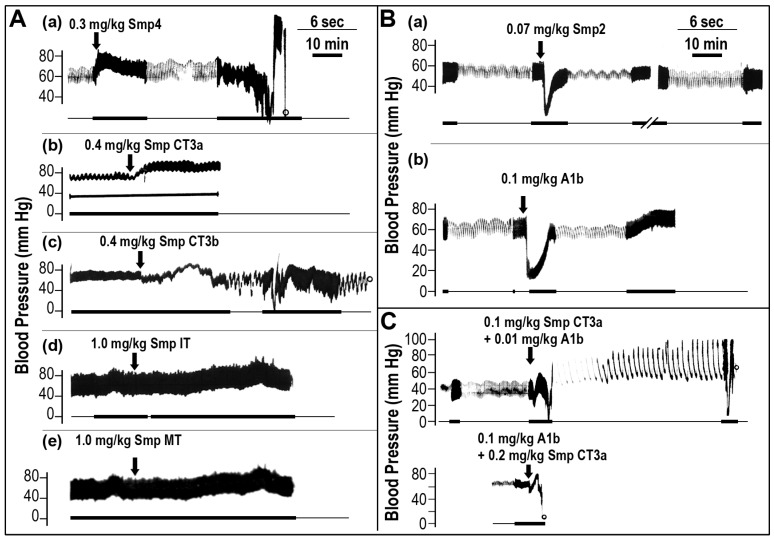
The effect on blood pressure of *Scorpio maurus palmatus* venom protein fractions isolated by gel permeation and purified toxins. (**A**) Neurotoxins effect on blood pressure; (**B**) PlA_2_ effect on blood pressure; (**C**) reconstitution of whole venom effect and synergistic effect on blood pressure of mixed doses of neurotoxin and PlA_2_ at different protein ratios. Each trace represents a single rat receiving a single dose of tested compound (representing measurements of 6–8 rats). The length of the horizontal lines represents the time course of the experiment: thin line (sec); thick line (min).

In the next step, we evaluated the effects of the isolated fractions on the arterial blood pressure of the rats (Figure 3). Fractionation of the venom revealed that the hypertensive and hypotensive effects resulted from different venom protein fractions. The hypertensive activity was attributed to the neurotoxic fraction, Smp4 (Figure 3A-a). This fraction not only increased the blood pressure, but also caused respiratory problems, mimicking the whole venom effect. In the past, several toxins were isolated from this fraction [9], and therefore, we used them to further investigate their potential hypertensive activity. While CT3a neurotoxin at a concentration of 0.4 mg/kg elicited a clear hypertensive effect (Figure 3A-b), which spontaneously returned to normal value after 90 min, the neurotoxin, CT3b, elicited respiratory perturbations with instability of animal blood pressure (Figure 3A–C). The neurotoxin for mice, named mammal toxin (Smp MT) [9], and the toxin to insects (Smp IT) were found ineffective in perturbation of rat blood pressure up to high doses of 1mg/kg (Figure 3A-d,e), although for Smp MT, symptoms of muscle paralysis were observed. The hypotensive response of the venom was only reproduced by the PlA_2_ containing fraction at a dose of 0.07 Smp2 (Figure 3B-a) and at 0.1 mg/kg for the purified PlA_2_-A1b enzyme (Figure 3B-b) [9], respectively. The Smp3 fraction, characterized by cytotoxic and cytolytic activity, was not effective in modulating blood pressure up to 1 mg/kg.

It was shown previously that the addition of the purified A1b-PlA_2_ enzyme elevated the lethal potency of the neurotoxins [9]. Since the *Scorpio maurus palmatus* venom biphasic effect on blood pressure is due to both the PlA_2_ and neurotoxin fractions, we tested qualitatively whether a combination of the purified A1b-PlA_2_ enzyme and Smp CT3a neurotoxin can mimic the dual effect of the venom on blood pressure, upon concomitant injection at a non-toxic, very low dose. While injected alone at low doses, the purified A1b-PlA_2_ enzyme (0.01 mg/kg) and the hypertensive factor CT3a (0.1 mg/kg) were found non-toxic and did not alter the blood pressure parameters. However, injection of their mixture at similar very low doses elicited a biphasic effect on blood pressure, followed by respiratory problems and animal death, mimicking the effect of the whole venom (Figure 3C-upper part). This effect was also observed at a higher dose ratio of A1b-PlA_2_ enzyme and the hypertensive factor, CT3a (Figure 3C-lower part).

### 2.3. The Effect of Venom and Isolated Protein Fractions on Histamine Release from Mast Cells

**Figure 4:**
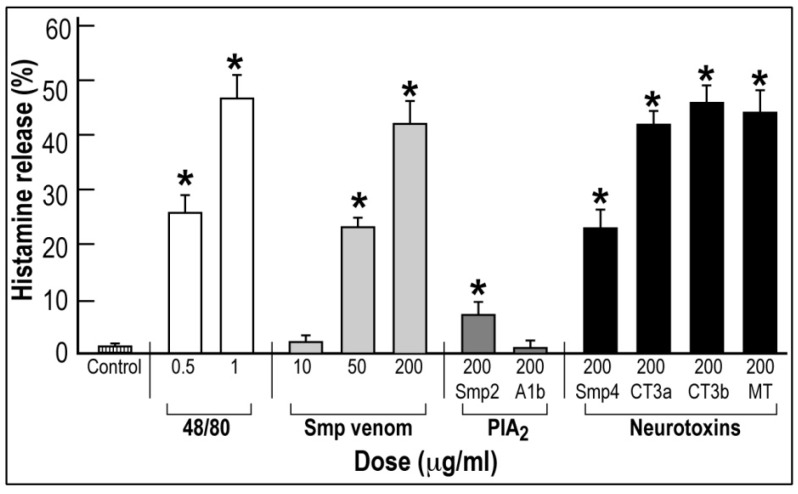
The effect of *Scorpio maurus palmatus* venom and protein fractions isolated by gel permeation and purified toxins on histamine release *in vitro* from mast cells.

Using the *in vitro* assay of histamine release from peritoneal mast cells, we evaluated the ability of *Scorpio maurus palmatus* venom and isolated fractions to release histamine by comparison to compound 48/80. Figure 4 clearly indicates that the *Scorpio maurus palmatus* whole venom induced histamine release in a dose-dependent manner, the effect of 50 µg/mL having been equivalent to that of a dose of 0.5 µg/mL of compound 48/80. Microscopic observation of the cells upon venom treatment, revealed typical degranulation and an increase in membrane permeability, as evidenced by the uptake of trypan blue (data not shown). 

The fraction Smp2 and derived A1b-PlA_2_ have shown only a very low effect on histamine release. Smp4 fraction and derived neurotoxins, CT3a, CT3b and MT, at 200 µg/mL caused histamine release similar to a dose of 1 µg/mL of compound 48/80 and with a higher specific activity than the whole venom. 

These results indicate that the histamine-induced released effect of the whole venom is produced *in vitro* by the Smp4 neurotoxic fraction and derived neurotoxins.

### 2.4. Discussion

The present study describes the complex effect of *Scorpio maurus palmatus* venom on rat blood pressure and identifies the hypertensive and hypotensive factors responsible for the biphasic effect of the whole venom. The present study unambiguously identified the phospholipase A_2_ protein fraction, Smp2, and derived phospholipase A1b [9] as responsible for this hypotensive effect. Phospholipases A_2_ were not found in Buthidae venoms; however, they are present in many snake venoms, which induces similar transient hypotensive effects on blood pressure [11]. The suggested mechanism of their action involves production of vasodilators and activation of a cyclooxygenase-dependent system [12,13]. Interestingly, from the point of view of scorpion venom pharmacology, the *Scorpio maurus palmatus* venom evolution regarding hemodynamics effects on blood pressure resembles evolutionary aspects of snake’s venoms. Since it has been reported that scorpion venoms induce mast cell degranulation and histamine release [14,15] and since histamine by activating H1 histamine receptor on endothelial cells [16] and generating nitric oxide produces vasodilation and fast hypotensive effects, we sought to investigate if the hypotensive effect of *Scorpio maurus palmatus* venom and isolated PlA_2_ is mediated by histamine release *in vitro* (Figure 4) and *in vivo* (data not shown). Indeed, the *Scorpio maurus palmatus* venom similar to the reference compound 48/80 induced a dose-dependent degranulation and histamine release from mast cells. Surprisingly, this effect was not mediated by PlA_2_, but was induced by all neurotoxins originally isolated from the neurotoxin fraction, Smp4. This effect *in vitro* may be attributed to the high isoelectric point of these toxins inducing nonspecific *in vitro* degranulation and histamine release from mast cells [17]. Furthermore, this effect of Smp4 neurotoxic fraction observed *in vitro* could not be reproduced *in vivo*, since no histamine was released in the blood of rats injected with Smp4 fraction at a dose of 200–500 µg/kg (data not shown). Therefore, we may hypothesize that the histamine induced release *in vitro* by neurotoxins is not relevant for the *in vivo* effects on blood pressure, and most probably, the venom effect is mediated *in vivo* by other unknown factors/mechanisms requiring future identification.

Our study also clearly identifies the neurotoxic Smp4 fraction as responsible for the hypertensive effect of the whole venom. This fraction contains several very basic and low molecular weight (31–34 amino acids) polypeptides, which were found to be relatively neurotoxic to different groups of animals, such as insects, crustacean and mammals [7,8,9]. From this series of neurotoxins, only CT3a and CT3b, previously defined as neurotoxins affecting crustacean [9], produced a clear hypertensive effect. Surprisingly, the other neurotoxins, including the mammal toxin, did not affect rat blood pressure (Figure 3). It is well established that mammal toxins from Buthidae venom interact with ionic channels of excitable cells, leading to a massive release of neurotransmitters. Voltage-gated Na+ channel toxins are mainly responsible for the toxic effects of scorpion envenomation and hypertensive effect on blood pressure [18,19]. The structure of the *Scorpio maurus palmatus* neurotoxins is unknown and, therefore, cannot be classified yet to either α or β scorpion toxins [18]. However, their similarity in amino acid composition to Buthidae mammal toxins [8] and ability to induce flaccid paralysis in albino mice upon cranial intraventricular injection at doses of 150–300 ng/kg, resembling the effects of *Tityus serrulatus* β scorpion toxin, strongly suggest that Smp neurotoxins CT3a and CT3b may also induce the hypertensive effect by activation of the voltage-sensitive sodium channel followed by catecholamine release, responsible for the peripheral blood vessel vasoconstrictor effect and the increase in blood pressure. Furthermore, CT3a polypeptide acted cooperatively with the A1b phospholipase, at nontoxic concentrations, mimicking the venom effect with respect to the biphasic modulation on blood pressure and lethality (Figure 3C). These findings may suggest that the previously noticed synergistic effect of *Scorpio maurus palmatus* venom toxins [7,9] is also reproduced at the level of the complex hemodynamic interaction between the venom components on blood pressure.

The regulation of blood pressure is mediated by heart activity, kidney, adrenal, lung, vascular system, baroreceptors, brain medulla cardiovascular centers and blood biochemistry. Therefore, in order to pinpoint the precise mechanisms by which *Scorpio maurus palmatus* venom and derived toxins are perturbing the blood pressure is very important to first study their effects on these different physiological systems before agreeing on a pharmacological mechanism contributing to their complex effect on blood pressure. Clinicians dealing with the stabilization of the blood pressure in the envenomed patient, in particular children, advocate administration of antiserum therapy [6]. Since the *Scorpio maurus palmatus* venom effect on the blood pressure is very fast, before most patients are brought to a medical facility, and since the pharmacokinetics of this venom components are unknown and no serotherapies are available, the only way to stabilize the hemodynamic failure following *Scorpio maurus palmatus* envenomation is a pharmacological approach employing conventional autonomic drugs, such as dobutamine [4], clonidine [20], prazosin [21] and other drugs, according to the time course of appearance and type of blood pressure pathophysiology.

## 3. Experimental Section

### 3.1. Materials

The *Scorpio maurus palmatus* venom was obtained by “electrical milking” of field-collected scorpions and stored lyophilized until use [8]. Histamine and histamine releasing factor 48/50 were purchased from Sigma (St. Louis, USA). 

### 3.2. Venom Separation by Gel Filtration

The extraction of the venom of the scorpion *Scorpio maurus palmatus* and the column chromatography procedures were used as previously reported [7,9]. Fractions with phospholipase A_2_ activity (Smp2 and A1b) and neurotoxins to different groups of animals (Smp4), crustacean toxins (CT) and a mammal toxin (MT) were obtained according to Lazarovici *et al.* [7,9] and Lazarovici and Zlotkin [8].

### 3.3. Blood Pressure Measurement

Adult rats of the Wistar strain (200–250 g) were used for these experiments. The rats had free access to water and standard chow until the day of the experiment. Experiments and animal care were approved by the Committee of Ethics of The Hebrew University and were performed in strict accordance with the Guide for the Care and Use of Laboratory Animals published by the US National Institutes of Health. Repeated administration of anesthesia was induced with pentobarbital sodium (50 mg/kg) intraperitoneally (i.p.), followed by tracheotomy and artificial ventilation, and body temperature was kept at 37–38 °C by a thermostatically controlled heating pad. Animals were ventilated with room air at 35–45 breaths/min supplemented with O_2_. At the end of the experiments, we confirmed that the arterial blood pH, pCO_2_ and bicarbonate were within physiological ranges using an ABL 80 Flex-pH/Blood Gas Analyzer. For blood pressure recording, a femoral artery was cannulated and connected to a Narco Bio-System, RP-1500 pressure transducer. A Narco Bio-System DMP-4B physiograph was used to record blood pressure. The tested substances were injected in a single dose per rat, and experiments were repeated in 8 rats (*n* = 8) in a volume of 0.2–0.4 mL in heparinized saline via the cannulated femoral vein. The mean arterial pressure was calculated using the following formula: (diastolic + (systolic − diastolic)/3). The results presented are from separate rats.

### 3.4. Histamine Release Assay

Mast cells were collected from the peritoneal perfusion with Tyrode from male inbred Wistar rats (200–300 g). Aliquots of 0.5 mL of 5 × 10^4^ peritoneal mast cell suspensions were equilibrated at 37 °C in Tyrode solution (137 mM NaCl, 2.7 mM KCl, 0.4 mM NaH_2_PO_4_, 1.4 mM CaCl_2_, 10 mM Hepes, 5.6 mM glucose, pH 7.4). Half milliliter of the tested compound dissolved in Tyrode and equilibrated at 37 °C was added to the above cell suspension, and incubation was carried for 30 min at 37 °C. The reaction was terminated by adding 1.5 mL of ice-cold calcium free Tyrode solution. The cells were isolated by centrifugation at 1,000 × *g* for 5 min and the supernatants collected. The pellets were resuspended in 2.5 mL Tyrode solution, placed for 10 min in a boiling water bath to release their residual histamine and centrifuged at 1,000 × *g* for 5 min. Aliquots from these supernatants were also collected. Histamine was assayed fluorometrically according to the method of Shore *et al.* [22]. Histamine release was calculated as a percentage of the total histamine content in each sample and subtracting the histamine released spontaneously (not exceeding 7%) [23]. Lytic damage and cell degranulation was confirmed by microscopic observation and trypan blue staining [10]. 

### 3.5. Phospholipase A_2_ Activity Measurement

Phospholipase activity was determined by a potentiometric titration (0.1 M NaOH) of acidity resulting from the release of fatty acids from a 1% suspension of phosphatidylcholine in the presence of 0.1% deoxycholate and 0.05 M CaCl_2_ at pH 8.0 and 37 °C, according to established methods [9]. The initial reaction velocity was determined, and the activity unit was defined as the amount of enzyme preparation releasing 1 µmol of fatty acids per minute.

### 3.6. Toxicity

Toxicity to albino mice (12–20 g) on arthropods and insects was determined by subcutaneous injection, as previously described [7,8,9].

### 3.7. Statistics

The blood pressure results are presented as the mean ± SD of three to six independent experiments (*n* = 6–18 rats) and were evaluated using the InStat3 statistics program (GraphPad, La Jolla, CA, USA). The histamine release results are presented as the mean ± SD of three independent experiments (*n* = 12). Statistically significant differences between experimental groups were determined by the Student's *t*-test and ANOVA, followed by the Bonferroni post-test and were considered significant when *p* ≤ 0.05.

## 4. Conclusions

*Scorpio maurus palmatus* venom upon injection into the femoral vein of anesthetized rats produced complex effects on the blood pressure. Using chromatographic separation of the venom and monitoring the femoral arterial pressure upon injection of the isolated proteins, it has been found that the hypotensive effect of the venom is caused by the PlA_2_ fraction and derived purified enzyme, while the hypertensive effect is caused by the neurotoxic fraction and purified crustacean neurotoxins. The whole venom effect on blood pressure was mimicked by mixing samples of the isolated hypotensive and hypertensive factors. The pharmacological mechanisms responsible for these effects are yet unknown and deserve clarification in order to design a therapeutic strategy for stabilization of the blood pressure in the envenomed patients.
